# Impact of Scar on Quality of Life in Well‐Differentiated Thyroid Carcinoma: A Systematic Review

**DOI:** 10.1002/oto2.70155

**Published:** 2025-09-19

**Authors:** Aníbal Ariza, Alvaro Sanabria

**Affiliations:** ^1^ Department of Surgery, School of Medicine Pontificia Universidad Javeriana Bogotá Colombia; ^2^ Department of Surgery, School of Medicine Universidad de Antioquia Medellín Colombia; ^3^ CEXCA, Centro de Excelencia en Enfermedades de Cabeza y Cuello Medellín Colombia

**Keywords:** quality of life, remote access thyroidectomy, scar, thyroid cancer, thyroidectomy

## Abstract

**Objective:**

Remote access techniques in thyroid surgery have been developed to improve cosmetic outcomes, based on the presumed impact of surgical scars on patients' quality of life (QoL). This systematic review aimed to evaluate the impact of surgical scars on the overall QoL of patients who have undergone thyroidectomy for low‐risk thyroid carcinoma. The review focuses on the rank of scar‐related QoL issues relative to other aspects assessed by validated QoL instruments.

**Data Sources:**

MEDLINE, EMBASE, CENTRAL, LILACS, Google Scholar (no language or time restrictions).

**Review Methods:**

A systematic review was conducted for original articles using validated QoL instruments with scar‐related domains in thyroid cancer patients who had undergone OT. Study quality was assessed using the JBI critical appraisal tool for cross‐sectional studies. Data were analyzed from 14 patient groups across 9 studies, representing 3658 patients.

**Results:**

In 12 of 14 samples, scar‐related issues ranked lower than sixth place in QoL assessments, with 7 samples placing scar‐related items at the lowest position. The Thyca‐Qol questionnaire was the most commonly used tool, though most studies had limitations, including selection and recall biases. However, across geographically and culturally diverse populations, scar‐related concerns consistently ranked lower in importance compared to other QoL factors.

**Conclusions:**

The impact of surgical scars on QoL in thyroid cancer survivors is low, with scar‐related items ranking among the least significant issues. Remote access techniques designed primarily for cosmetic outcomes should demonstrate additional benefits to justify their use.

Thyroid carcinoma is one of the most common cancers worldwide and overdiagnosis has contributed to a rise in its incidence.^1^ Open thyroidectomy (OT) remains the most used therapeutic approach, with minimal morbidity and excellent oncologic outcomes when performed by experienced surgeons. It is estimated that over 150,000 thyroidectomies are performed in the United States each year.^2^

To reduce scar visibility in the neck, the standard open approach involves making an anterior transverse incision along a natural skin line. Remote access techniques (RAT) in thyroid surgery have recently emerged, which have demonstrated comparable safety and oncologic outcomes to the conventional approach.^3^ The major goal of these treatments was to reduce the aesthetic impact of the scar, and several authors support their usage based on the importance of the cosmetic outcome to the patient's quality of life (QoL).^3^ However, the stated assumption as a reason for using these techniques in thyroid cancer patients has yet to be evaluated.

Cosmetic results are believed to be an essential element in adopting RAT procedures, although they relate to increased expenditures to the health system, particularly in developing nations.^4^ As a result, we decided to conduct a systematic review to evaluate the influence that surgical scars have on the QoL, as well as their rank within the items included in specific instruments that are utilized to measure the QoL in people who have been diagnosed with thyroid cancer.

## Materials and Methods

This systematic review was developed according to the Cochrane Collaboration recommendations^5^ and is reported according to the PRISMA 2020 guidelines.^6^ Prior to this, the protocol was registered in the PROSPERO registry (CRD42023205733).

Because this study did not use patient data, it did not require authorization from the IRB.

### Eligibility Criteria

#### Studies

We included original studies that used validated tools to assess QoL in thyroid cancer patients after an OT. These instruments should contain a particular item or domain associated with the scar's effect. Only studies that reported specific values of each item within the specified instrument were included in the analysis.

##### Patients

Patients who had either a partial or total OT due to low‐risk thyroid carcinoma.

##### Interventions

The evaluation of QoL after OT.

#### Outcomes

The primary outcome was the rank of the scar or cosmesis‐related item in the overall ranking of QoL items. The clinical and methodological heterogeneity of the included studies precluded a meta‐analysis. QoL meta‐analyses often treat overall scale values as continuous variables, which is not universally accepted given population heterogeneity. Furthermore, synthesizing individual items or domains on a global scale lacks statistical and methodological reliability. Because this study is focused on the scar item, its major goal was to determine its relative ranking on the total scale, making a meta‐analytic technique inappropriate.

#### Search Strategy

The authors did an independent search of the papers using MEDLINE, EMBASE, CENTRAL, LILACS, and Google Scholar. There were no time or language restrictions. The last search was conducted in January 2024. A supplementary table provides a detailed description of the search strategy. Initially, the articles abstracts were reviewed, and the most suitable ones were selected. These were then examined and chosen based on the established inclusion criteria. The disagreements were resolved by consensus. We used a snowball method to find additional articles based on the references of chosen studies. The flowchart was built with Covidence software. (Available at www.covidence.org.).

### Data Collection

The authors independently reviewed the entire texts of the chosen articles and completed an online form. The discrepancies between the 2 data sets were resolved by reevaluating the original paper and achieving consensus. Four of the studies included clinically heterogeneous groups (total versus partial thyroidectomy; RAI use and follow‐up duration), therefore the assessment of QoL might differ. As a result, each sample was treated independently in the analysis.

### Variables

The data collecting form included the number of patients, the design of the study, the thyroidectomy extension, the QoL assessment instrument and the mean value of each of the instrument item or domains.

### Risk of Bias Assessment

The authors evaluated chosen papers independently using the JBI critical evaluation criteria for cross‐sectional research (https://jbi.global/critical‐appraisal‐tools). The JBI Checklist for Analytical Cross‐Sectional Studies evaluates cross‐sectional study methodology. It evaluates research design using 8 criteria: clearly specified inclusion criteria, full participant descriptions, accurate and reliable exposure and outcome measurement, confounding factor identification and control, and suitable statistical analysis. Each item is scored “Yes,” “No,” “Unclear,” or “Not Applicable,” assessing research quality. Differences were resolved via consensus.

### Analysis

After collecting the mean value of each item evaluated on the primary study, we ranked the items from highest to lowest score, with higher scores indicating a greater impact on QoL. Because the ranking procedure provides information on the relative importance of each item within the sample but does not indicate the magnitude of this effect (if despite being in a low ranking, it has a high score) we decided to calculate the mean absolute difference (MAD) between the scar item's absolute score and the absolute scores of the first and last items on the QoL scale. The MAD assesses the scar's relative importance in the overall context of QoL, as well as its proximity to other items commonly associated with poor QoL. To assess if the MAD had a clinically significant difference with respect to the highest‐scoring item, Norman's criteria was used, which states that a difference greater than 0.5 SD is significant.^7^ No subgroup analysis was planned.

## Results

### Study Characteristics

**Figure 1:**
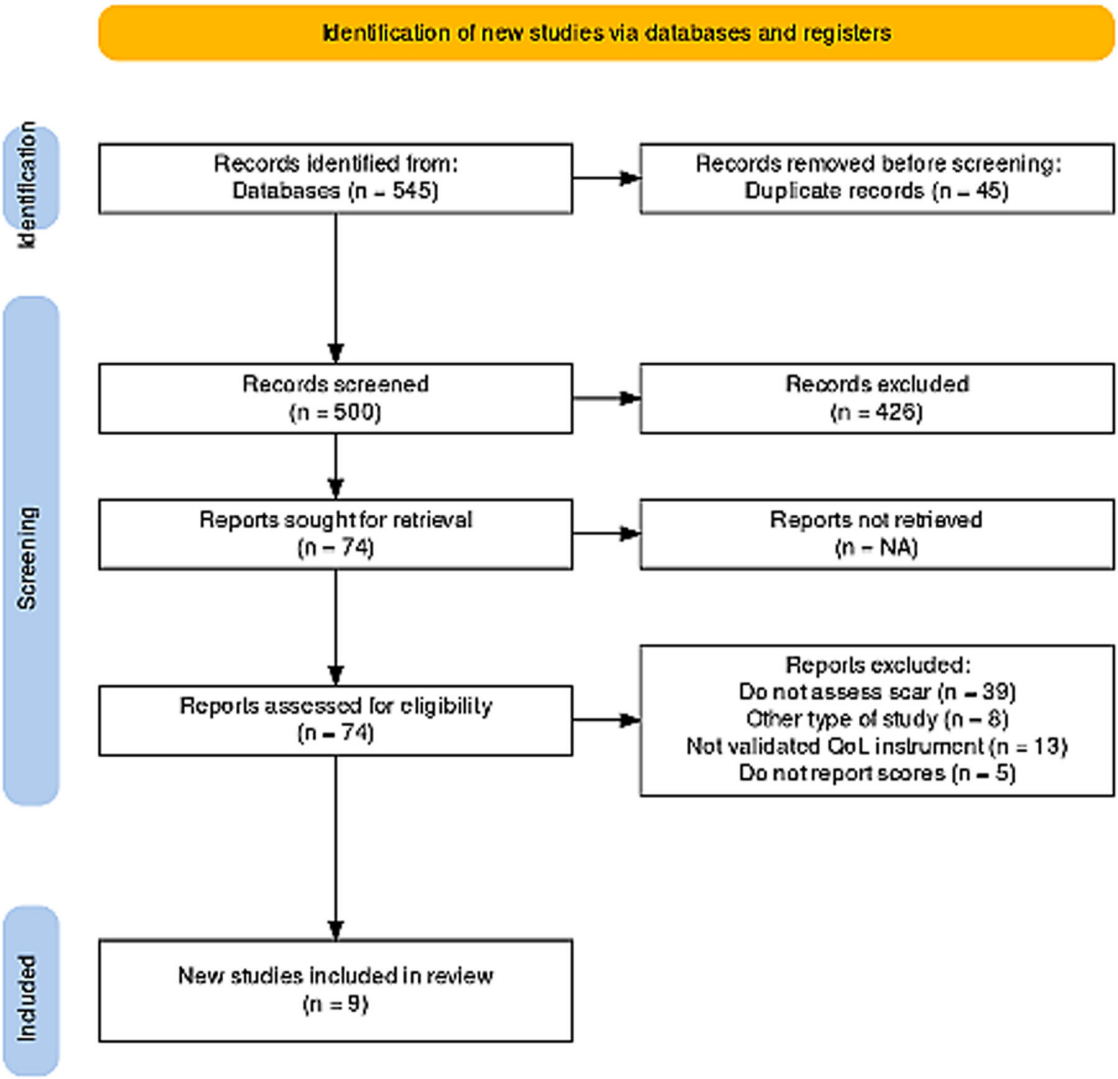
Flow chart of included studies in systematic review of scar impact in quality of life.

The systematic review evaluated 545 studies, with only 9 full‐text articles judged appropriate Figure 1. Four of the studies included clinically heterogenous groups, where the assessment of the QoL may differ. (Husson et al,^8^ included 3 subgroups based on the period between diagnosis and evaluation, Goldfarb et al,^9^ 2 groups based on participant age, Chen et al, 2 groups based on the extent of thyroidectomy, and Ahn et al,^10^ 2 groups based on the use of RAI.) As a result, we collected data of 14 subgroups and each subgroup was analyzed independently. Table 1 summarizes the most significant findings from the included studies. Except for one prospective cohort research, all the studies used a cross‐sectional design. A total of 3658 patients were included. Most of them were Asian (1894 patients, 51%), while the proportion of women ranged from 72% to 91%.

**Table 1:** Characteristics of Included Studies

Author, year	Country	Time from diagnosis to QOL	Number of patients	Histology	Age (mean ± sd /median (range)	Sex (female)	Thyroidectomy extension	Neck dissection	Design
Husson et al 2013	Netherlands	<5 years	81	95% PAP/FOL 4% MED	56.4 ± 14.5	75%	NE	NE	Cross‐sectional
		5‐10 years	86						
		>10 years	137						
Goldfarb et al 2016	USA	Young adults 78% >1 year	751	97% PAP/FOL 3% MED	62% >40 years	89%‐93%	NE	NE	Cross‐sectional
		>40 y 74% >1 year	277						
Rogers et al 2017	United Kingdom	2.6 y (IQR 1.6‐3.8)	169	76% PAP 22% FOL	51 (40‐63)	72%	NE	NE	Cross‐sectional
Jeon et al 2019	Korea	3.2 y (IQR 2.1‐4.4)	148	100% PAP	51 ± 10.4	85%	TT	NE	Cross‐sectional
Ahn et al 2020	Korea	TT 3.5 y (IQR 2.4‐4.8)	107	100% PAP	54.2 ± 8.9	90%	TT	NE	Cross‐ sectional
		TT + RAI 3.8 y (IQR 2.9‐4.5)	182		52.9 ± 9.4	82%			
Lan et al 2020	China	1.7 ± 1.4 y	34	100% PAP	42.4 ± 9.8	91%	PT/TT	NE	Cross‐ sectional
Chan et al 2021	Hong Kong	64.6% >5 years	613	79.8% PAP 10.9% FOL	53 (18‐91)	80%	91.4% TT 8.6% PT	14.8%	Cross‐sectional
Chen et al 2022	China	TT 1 year	361	100% PAP/FOL	37 (31‐47)	76.7%	TT	71.2%	Prospective cohort
		PT 1 year	419		37 (31‐45)	76.8%	PT	49.6%	
Gomez et al 2024	Colombia	64% <2 y	293	96.6% PAP	47 ± 14.2	84%	75% TT 25% PT	37.9%	Cross‐sectional

Abbreviations: FOL, follicular carcinoma; IQR, interquartile range; MED, medullary carcinoma; NE, not specified; PAP, papillary carcinoma; PT, partial thyroidectomy, TT, total thyroidectomy.

The Thyroid Cancer‐Specific Quality of Life Scale (Thyca‐Qol) was the only validated assessment tool that provided means for each questionnaire item. The remaining studies included other instruments that did not provide the necessary data and were excluded.^11^ The Thyca‐Qol questionnaire has 24 questions divided into 7 categories (neuromuscular, voice, concentration, sympathetic, throat/mouth, psychological, and sensory) and 7 single items. Each item contains 4 response categories (1 “not at all,” to 4 “very much”) with a time limit (1 week for most items, 4 weeks for sexuality questions). These scores are converted to a 0 to 100 scale. A higher score indicates a poorer outcome; only the item of sexual interest has a differential evaluation, with higher scores indicating a better outcome. The outcomes were ordered from 1 to 13, highest to lowest.

### Outcomes

Husson et al,^8^ evaluated the QoL of 304 patients. In the groups of survivors with 5 to 10 years and >10 years of follow‐up, problems with scars obtained the lowest scores, with a MAD of 17.7 and 15.4 with the first item (felt chilly), respectively. Among survivors with less than 5 years of follow‐up, scar problems ranked 10th, with a MAD of 15.2 for the greatest (felt chilly) and 3.3 for the lowest (gained weight). Goldfarb et al^9^ assessed the QoL of thyroid cancer after 1 year. The authors conducted age‐specific analyses (<40 vs ≥40). Scar problems rated last among 751 patients over the age of 40, with a MAD of 31.7 when compared to the top item (neuromuscular). In the 277 patients under the age of 40, the scar was likewise positioned in the 10th place with a MAD of 16.6 and 10.96, respectively, for the highest item (cold sensation) and the lowest item (voice).

Rogers et al,^12^ assessed the QoL in 169 patients from the United Kingdom. The scar item placed 13th, with a mean score of 13, and a MAD of 19 in comparison with the most significant item (neuromuscular). Jeon et al,^13^ studied 148 Korean patients, with a 38‐month interval between diagnosis and QoL assessment. Scar scored last in this sample, with a score of 34.1 and a MAD of 16.56, with the highest score being tingling hands/feet. Ahn et al,^10^ evaluated QoL in Korean patients treated with total thyroidectomy (TT) alone or with radioactive iodine (RAI) and a median interval between therapy and questionnaire completion longer than 3.5 years. The scar item in the TT alone category rated ninth, with a mean score of 38.55. The MAD for the highest item (tingling hands/feet) and the lowest item (felt chilly) was 10.28 and 3.97, respectively. In the TT + RAI subgroup, the scar was placed twelfth. Chan et al,^14^ followed 613 survivors for more than 5 years. In this study, the scale scores were not adjusted to a scale of 0 to 100. As a result, we opted to do this normalization using the available data. The highest score was given for tingling in the hands and feet. The scar‐related item was rated 11th, with a MAD of 48.5, compared to the item with the highest score. Gómez et al,^15^ published a study with a Latin American population. They included 293 Colombian patients, with the majority undergoing TT. Scar‐related problems were at the bottom, with a MAD of 23.3 compared to the top item (headache). Lan et al,^16^ conducted the research in 34 Korean patients with a 15‐month median time frame from therapy to QoL evaluation. The scar item was rated seventh. The MAD for the top item (psychological) and the lowest item (tingling in hands/feet) was 15.69 and 7.84, respectively.

Chen et al,^17^ presented the only prospective trial to date, with 1060 patients treated with lobectomy or TT and followed for 1, 3, 6, and 12 months. This study included 2 subgroups: 361 patients treated with lobectomy and 419 with TT who were assessed at 12 months. The outcomes of these patient groups differ from those stated above. The scar in both groups was in the second place, with a MAD for the top item (psychological) of 10.4 and 12.5 and with the last item (chilly) of 14.24 and 16.25 for lobectomy and TT, respectively. A careful examination of the additional material from this article (Supplemental eTable 9, available online)^17^ reveals that the average of items other than the scar, such as voice, concentration, and sympathetic, was too low, but we couldn't find a clinical reason to explain these findings.

**Figure 2:**
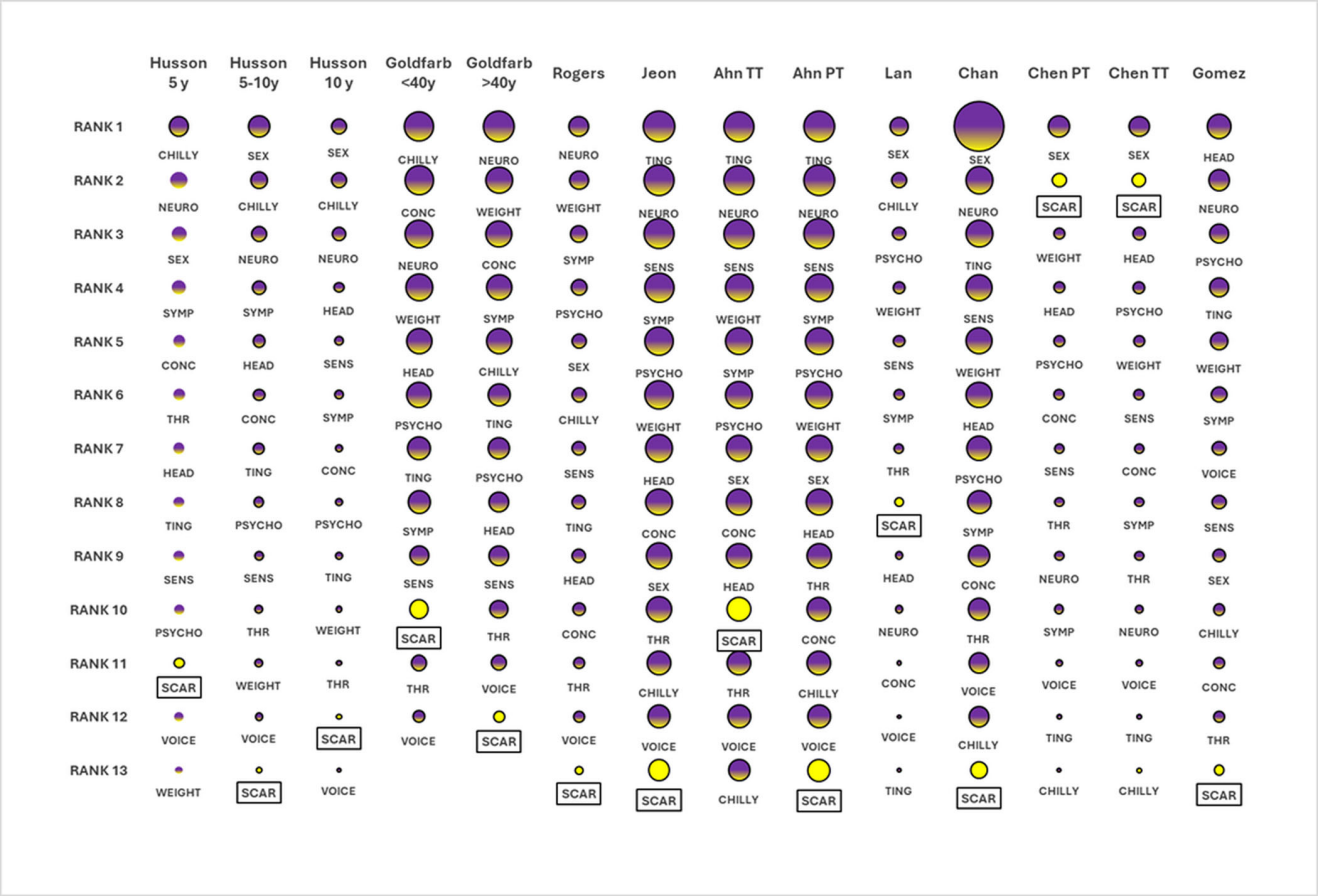
Heat chart showing the rankings of the ThycaQol domains and individual items. The bubble size is equivalent to the scale score. The scar item is shown in light color.

In 12^8^, ^9^, ^10^, ^12^, ^13^, ^14^, ^15^, ^16^ of the 14 previously mentioned patient groups (79% of patients), the scar item consistently rated lower than sixth. In 7 of these samples, scar had the least significant influence on QoL. Figure 2 and Supplemental Table S1, available online. Eleven (3070 patients, 84%)^8^, ^9^, ^10^, ^13^, ^15^ of the 14 populations examined found that the difference in scores between the highest‐ranking item and the scar item fulfilled Norman's criteria, implying that the variations in scores are clinically significant.

In 7^8^, ^9^, ^10^, ^13^, ^14^, ^15^, ^17^ out of 9 studies, the methodological quality is thought to be at moderate or low risk of bias Supplemental Table S2, available online. The most significant weaknesses were a lack of methods (subgroup analysis, regression analysis) for adjusting QoL scores for known characteristics (age, extent of surgery, etc.), which might change the ranking of items. Furthermore, deficiencies in patient selection criteria were discovered, resulting in clinically different populations that cannot be included in a meta‐analytic synthesis. However, the scar item's position in high‐risk of bias studies^12^, ^16^ and the majority of moderate/low‐risk studies^8^, ^9^, ^10^, ^13^, ^14^, ^15^, ^17^ was similar.

## Discussion

OT is the most common treatment for thyroid cancer globally. With the continually growing frequency of thyroid cancer, it is unsurprising that thyroidectomies will continue to rise.^1^ Billroth and Kocher made substantial advancements to thyroid surgery at the beginning of the twentieth century, reducing postoperative complications and death rates.^18^ As the safety of thyroidectomy improved, the surgical paradigm was modified to improve QoL and to meet patient's expectations for a more cosmetic outcome. This resulted in improvements to the open approach and the development of RAT aimed at avoiding scarring in the anterior portion of the neck.^19^ The major goal of these RATs is to improve cosmetic results. Although these techniques have shown safety, efficacy, and reproducibility, there are concerns about the need for more extensive dissections to access the thyroid gland, making it more invasive; the reports of increased postoperative pain and longer operative time, and the emergence of complications inexistent in the open method, such as mental anesthesia and tracheal rupture.^20^, ^21^

Culture, religion, and ethnicity may all impact how people perceive their scars. The significance of the postoperative scar in the Eastern population has frequently been linked to a negative social impact, which may explain the importance of surgical approaches that avoid visible scars, as well as the greater development of these techniques in Asian countries. However, scientific data proving that a postthyroidectomy surgical scar restricts social contact or is linked with considerable functional impairment and intolerable psychological load is limited. Alci et al^22^ investigated the perception of thyroidectomy scar features and their possible influence on appearance, self‐confidence, attractiveness, and self‐perception in patients without thyroid illness. When compared to Turkish respondents, Korean individuals reported a higher perception of damage (53% vs 25%) and a stronger unfavorable impact on their self‐image (17% vs 8%).

Several instruments have been created to assess the QoL in thyroid cancer patients,^23^ but few have undergone rigorous psychometric validation. The ThyCa‐HRLQOL, EORTC‐Thy34, MDASI‐Thy, and Thyca‐Qol are QoL assessment tools recommended for thyroid carcinoma survivors.^24^ They assess psychological and functional complaints, symptoms, and psychosocial concerns. Thyca‐Qol is the most widely used tool for assessing the QoL in thyroid cancer patients due to its methodological quality and condition specificity. Furthermore, it provides a unique item that allows an evaluation of the scar's impact in terms of global QoL. To our knowledge, there are no systematic reviews that examine the impact of scars in the global context of QoL and rate their rank. This study analyzed data from 3658 patients with thyroid cancer, to determine the influence of surgical scars on QoL total score, as well as its relative position among the most and least significant items.

In this study, we discovered that scar‐related issues appear to be of very low repercussions in terms of overall QoL for patients with thyroid cancer. Of the 14 samples analyzed, the scar‐related item was typically placed below sixth place in 12 groups, and it was regarded as the least impactful in 6 of them. The results revealed an average absolute difference of 16.4 (range 0‐48.5) points between the highest‐scoring item and the scar‐related difficulties. The time between diagnosis and the use of the ThycaQol scale is an essential consideration in scar evaluation. Although most research did not account for this factor, it is known that the effect of scarring diminishes over time. Husson et al,^8^ found no difference in scar item rating after considering this aspect in their study.

Most studies measured QoL one year or more after diagnosis, indicating that the findings represent the long‐term status rather than the short‐term situation. Goldfarb et al,^9^ conducted the only study to compare the impact of age on QoL (under 40 vs over 40). Although the scar item ranked higher in the younger cohort (tenth vs twelfth), it did not hold a dominant position in the overall ranking. Some authors argue that aesthetics is significant across ethnicities and that patients may prefer not to have a neck scar.^25^ Other studies examining the appearance of scars in head and neck procedures have found high levels of satisfaction, particularly 6 months following surgery.^15^ Although the postsurgical scar has been a major concern for thyroid surgeons due to its potential impact on QoL, it is well known that physicians and patients have different perspectives on neck and facial scars, and doctors may under or overestimate the impact of surgical scars on QoL.^26^ Several qualitative research examined the coping techniques that patients employ with the illness, as well as the impact of the scar left by the therapy.^27^ For some patients, the scar represents triumph over the sickness or satisfaction in having fought and survived. Furthermore, some patients see it as a sign of belonging to a group and an opportunity to establish relationships with others in similar circumstances.^28^ Several studies have found that most patients perceive the scar as just a temporary condition in the context of their illness.^29^, ^30^, ^31^ The possible influence of genetic susceptibility to hyperpigmentation and keloid development in Asian people is also a relevant factor. Several studies have shown different genetic variations and polymorphisms linked to enhanced fibroproliferative responses and pigmentation pathways in Asian people.^32^ These genetic variables might explain the observed disparities in wound healing outcomes, such as a greater proclivity for keloid development and hyperpigmentation. This study found that the scar item's rating in studies done in Asian (1864 patients, 51%)^10^, ^13^, ^14^, ^16^ and Western (1794 patients, 49%)^8^, ^9^, ^12^, ^15^ individuals was similar. Furthermore, the variation in ranks, even among the same Asian ethnicities, indicates the presence of an unexplained factor.

Most studies found significant differences between the highest‐scoring item and the scar‐ related item, with the exception of Chen et al,^17^ This last study included patients with a low to intermediate risk of recurrence and was the only one in which the scar‐related item was ranked high and other items with consistently high scores, such as felt chilly and tingling in the hands and feet, were ranked last. We found no explanation for these differences other than the research design and the higher use of neck dissection. As a result, our findings challenge the often‐used affirmation that aesthetic considerations have a major impact on QoL. Therefore, RAT for thyroidectomy must demonstrate other benefits beyond aesthetics to justify its application in public health systems.^20^

This study has some weaknesses that must be addressed. The Thyca‐Qol instrument includes only one scar‐related question, although it does not assess the scar's specific features such as length, thickness, color, or keloid development. These aspects could have an impact on the individual patient experience. There are even methodological limitations related to the psychometric characteristics of the scar item (lack of evaluation of convergent validity and test‐retest reliability). However, of all the specific instruments that evaluate QoL in patients with thyroid cancer, the Thyca‐Qol is the most widely evaluated and the one that offers the most detailed information.

The studies were influenced by selection and recall bias, given not all patients completed the questionnaires and the majority were collected sometime after surgery. However, the consistency of the results across samples, age ranges, and extended follow‐up periods shows that the impact of these biases may be minor. Most studies failed to adjust for variables such as patient age and gender, time between surgery and QoL assessment, surgery extension, and neck dissection, which may have influenced the results. However, Figure 2 shows that, despite variations in these factors, the ranking results are not significantly influenced. Unfortunately, due to the small number of studies, no subgroup analysis was done with significant clinical characteristics such as the extent of surgery, the addition of neck dissection, or the time of application of the ThycaQol questionnaire.

Finally, we acknowledge that esthetic and appearance issues following surgery are significant to patients who have had a thyroidectomy to treat thyroid cancer. However, present data do not support the premise that the scar item scores high in QoL questionnaires. Some patients place great importance on aesthetic results or have a history of hypertrophic scars, and surgeons must appreciate how patient preferences impact surgical decision‐making. This opens the door to the use of RAT in carefully selected patients. However, given the effects of longer surgical times and higher costs, the surgical community should assess the current arguments for the use of RAT, which suggests that its usage improves patients’ QoL by reducing scarring.

## Conclusion

In conclusion, this systematic review demonstrates that aesthetic results in thyroid cancer survivors do not rank high when compared to other aspects of their QoL. The scar‐related item was consistently found in the final position across many groups with geographical and cultural diversity. In addition to cosmetic reasons, the use of RATs should be justified by other clinical or individual outcomes (risk‐benefit, costs, availability) that are not often covered by existing QoL measurement methods.

## Author Contributions

**Anibal Ariza**, conceptualization, methodology, validation, investigation, data curation, writing—original draft and writing—review and editing; **Alvaro Sanabria**, conceptualization, methodology, validation, investigation, data curation, formal analysis, writing—original draft and writing—review and editing.

## Disclosures

### Competing interests

None.

### Funding source

None.

## Supporting information

Supporting information.

**Supplementary Table 1.** Ranking of QoL items in the Thyca‐Qol instrument†. SCAR: Scar problems; CHILLY: Felt chilly; HEAD: headache; PSYCH: Psychological, SENS: Sensory; WEIGHT: Gained weight; CONC: Concentration; SYMP: Sympathetic; THR: Throat/mouth; NEURO: Neuromuscular; TING: Tingling hands/feet; MAD 1: minimal absolute difference from the first ranked item to scar item; MAD2: minimal absolute difference from the scar item to the las t ranked item; CSD: clinically significant difference according to Norman rule.; NA: not applicable * Sex item was not reported. † item of sexual interest has a differential evaluation, with higher scores indicating a better outcome.

**Supplementary table 2.** Risk of bias assessment.
