# Supplementary material for: Impact of Scar on Quality of Life in Well‐Differentiated Thyroid Carcinoma: A Systematic Review
Source: OTO Open. 2025 Sep 19;9(3):e70155. doi: 10.1002/oto2.70155 (PMC12447349; doi:10.1002/oto2.70155)
Supplement: Supplementary file 1 — Supporting information. [file OTO2-9-e70155-s001.docx]

**Supplementary material**

**Search strategy:**

**Medline**

(((("cicatrix"[MeSH Terms] OR scar[Text Word])) OR ("quality of life"[MeSH Terms] OR quality of life[Text Word]))) AND ("Thyroid Neoplasms"[Mesh]) AND "Thyroidectomy"[Mesh])

**EMBASE**

('skin scar'/exp OR 'skin scar' OR 'quality of life') AND 'differentiated thyroid cancer' AND 'thyroidectomy'
