# Supplementary material for: Impact of Scar on Quality of Life in Well‐Differentiated Thyroid Carcinoma: A Systematic Review
Source: OTO Open. 2025 Sep 19;9(3):e70155. doi: 10.1002/oto2.70155 (PMC12447349; doi:10.1002/oto2.70155)
Supplement: Supplementary file 2 — Supplementary Table 1. Ranking of QoL items in the Thyca‐Qol instrument†. SCAR: Scar problems; CHILLY: Felt chilly; HEAD: headache; PSYCH: Psychological, SENS: Sensory; WEIGHT: Gained weight; CONC: Concentration; SYMP: Sympathetic; THR: Throat/mouth; NEURO: Neuromuscular; TING: Tingling hands/feet; MAD 1: minimal absolute difference from the first ranked item to scar item; MAD2: minimal absolute difference from the scar item to the las t ranked item; CSD: clinically significant difference according to Norman rule.; NA: not applicable * Sex item was not reported. † item of sexual interest has a differential evaluation, with higher scores indicating a better outcome. [file OTO2-9-e70155-s002.docx]

| **Author** | **Husson 2013** | **Husson 2013** | **Husson 2013** | **Goldfarb 2016 *** | **Goldfarb 2016 *** | **Rogers 2017** | **Jeon 2019** | **Ahn 2020** | **Ahn 2020** | **Lan 2020** | **Chan 2021** | **Chen 2022** | **Chen 2022** | **Gomez 2024** |
| --- | --- | --- | --- | --- | --- | --- | --- | --- | --- | --- | --- | --- | --- | --- |
|  | **< 5 y** | **5-10 y** | **>10 y** | **Young** | **>40 y** |  |  | **TT** | **TT + RAI** |  |  | **TT** | **PT** |  |
| **RANK 1** | **CHILLY 31.7±.32.9** | **SEX 35.6±25.8** | **SEX  24,4± 24.4** | **CHILLY 47.1±27.7** | **NEURO 49.9±27.1** | **NEURO 32** | **TING 50.6±22.3** | **TING 48.8±17.9** | **TING 50.4±20.6** | **SEX  29.4±24.3** | **SEX  81.5 ±16.5** | **SEX**  **34.3±24.7** | **SEX**  **33.2±23.6** | **HEAD 39.7± 36.5** |
| **RANK 2** | NEURO | CHILLY | CHILLY | CONC | WEIGHT | WEIGHT | NEURO | NEURO | NEURO | CHILLY | TING | SCAR 22.8±27.8 | SCAR 21.81±26 | NEURO |
| **RANK 3** | SEX | NEURO | NEURO | NEURO | CONC | SYMP | SENS | SENS | SENS | PSYCH | SENS | HEAD | WEIGHT | PSYCH |
| **RANK 4** | SYMP | SYMP | HEAD | WEIGHT | SYMP | PSYCH | SYMP | WEIGHT | SYMP | WEIGHT | WEIGHT | PSYCH | HEAD | TING |
| **RANK 5** | CONC | HEAD | SENS | HEAD | CHILLY | SEX | PSYCH | SYMP | PSYCH | SENS | HEAD | WEIGHT | PSYCH | WEIGHT |
| **RANK 6** | THR | CONC | SYMP | PSYCH | TING | CHILLY | WEIGHT | PSYCH | WEIGHT | SYMP | NEURO | SENS | CONC | SYMP |
| **RANK 7** | HEAD | TING | CONC | TING | PSYCH | SENS | HEAD | SEX | SEX | THR | SYMP | CONC | SENS | VOICE |
| **RANK 8** | TING | PSYCH | PSYCH | SYMP | HEAD | TING | SEX | CONC | HEAD | SCAR 13.7±20.3 | CONC | SYMP | THR | SENS |
| **RANK 9** | SENS | SENS | TING | SENS | SENS | HEAD | CONC | HEAD | THR | HEAD | VOICE | THR | NEURO | SEX |
| **RANK 10** | PSYCH | THR | WEIGHT | SCAR 30.5± 29 | THR | CONC | THR | SCAR 38.5±21.5 | CONC | NEURO | THR | NEURO | SYMP | CHILLY |
| **RANK 11** | SCAR  16.5± 26.9 | WEIGHT | THR | THR | VOICE | THR | CHILLY | THR | CHILLY | CONC | CHILLY | VOICE | VOICE | CONC |
| **RANK 12** | VOICE | VOICE | SCAR 8±20.1 | VOICE | SCAR 18.1±24.4 | VOICE | VOICE | VOICE | VOICE | VOICE | SCAR 33±16.5 | TING | TING | THR |
| **RANK 13** | WEIGHT | SCAR  9.1±23.3 | VOICE |  |  | SCAR 13 | SCAR  34.1±16.2 | CHILLY | SCAR 35.8±19.2 | TING | PSYCH | CHILLY | CHILLY | SCAR 16.38±26.3 |
| **MAD1** | 15,2 | 26,5 | 16,4 | 16,6 | 31,7 | 19 | 16,5 | 10,2 | 14,5 | 15,7 | 48.5 | 12.5 | 10.4 | 23,3 |
| **MAD2** | 3,3 | 0 | 0.7 | 10,9 | 0 | 0 | 0 | 3,97 | 0 | 7,8 | 5,5 | 16,2 | 14,4 | 0 |
| **CSD** | Yes | Yes | Yes | Yes | Yes | NA | Yes | Yes | Yes | Yes | Yes | No | No | Yes |
