# Supplementary material for: Impact of Scar on Quality of Life in Well‐Differentiated Thyroid Carcinoma: A Systematic Review
Source: OTO Open. 2025 Sep 19;9(3):e70155. doi: 10.1002/oto2.70155 (PMC12447349; doi:10.1002/oto2.70155)
Supplement: Supplementary file 3 — Supplementary table 2. Risk of bias assessment. [file OTO2-9-e70155-s003.docx]

| **Risk of bias assessment** | | | | | | | | | **Overall risk** |
| --- | --- | --- | --- | --- | --- | --- | --- | --- | --- |
| **Study** | Were the criteria for inclusion in the sample clearly defined? | Were the study subjects and the setting described in detail? | Was the exposure measured in a valid and reliable way? | Were objective, standard criteria used for measurement of the condition? | Were confounding factors identified? | Were strategies to deal with confounding factors stated? | Were the outcomes measured in a valid and reliable way? | Was appropriate statistical analysis used? |  |
| Husson 2013 | Yes | Yes | Yes | Yes | Yes | Yes | Yes | Yes | Low |
| Goldfarb 2016 | Unclear | Yes | Yes | Yes | Yes | Yes | Yes | Yes | Moderate |
| Rogers 2017 | Unclear | Yes | Yes | Yes | Yes | Unclear | Yes | Unclear | High |
| Ahn 2020 | Yes | Yes | Yes | Yes | Yes | Unclear | Yes | Yes | Moderate |
| Lan 2020 | Yes | Yes | Yes | Yes | Yes | Unclear | Unclear | Yes | High |
| Chan 2021 | Yes | Yes | Yes | Yes | Yes | Yes | Yes | Unclear | Moderate |
| Chen 2022 | Yes | Yes | Yes | Yes | Yes | Yes | Yes | Yes | Low |
| Jeon 2019 | Yes | Yes | Yes | Yes | Yes | Yes | Yes | Yes | Low |
| Gomez 2024 | Yes | Yes | Yes | Yes | Yes | Unclear | Yes | Yes | Moderate |
